# A Physicians' Wish List for the Clinical Application of Intestinal Metagenomics

**DOI:** 10.1371/journal.pmed.1001627

**Published:** 2014-04-15

**Authors:** Ingeborg Klymiuk, Christoph Högenauer, Bettina Halwachs, Gerhard G. Thallinger, W. Florian Fricke, Christoph Steininger

**Affiliations:** 1 Core Facility Molecular Biology, Center for Medical Research, Medical University of Graz, Graz, Austria; 2 Department of Gastroenterology and Hepatology, Medical University of Graz, Graz, Austria; 3 Institute for Genomics and Bioinformatics, Graz University of Technology, Graz, Austria; 4 Core Facility Bioinformatics, Austrian Centre of Industrial Biotechnology, Graz, Austria; 5 Department of Microbiology and Immunology, Institute for Genome Sciences, University of Maryland School of Medicine, Baltimore, Maryland, United States of America; 6 Division of Infectious Diseases, Department of Medicine I, Medical University of Vienna, Vienna, Austria

## Abstract

Christoph Steininger and colleagues explore how multiple infectious, autoimmune, metabolic, and neoplastic diseases have been associated with changes in the intestinal microbiome, although a cause-effect relationship is often difficult to establish. Integration of metagenomics into clinical medicine is a challenge, and the authors highlight clinical approaches that are of high priority for the useful medical application of metagenomics.

*Please see later in the article for the Editors' Summary*

**Summary Points**

- Multiple infectious, autoimmune, metabolic, and neoplastic diseases have been associated with changes in the intestinal microbiome, although a cause–effect relationship is often difficult to establish.
- Here we discuss the problems, applications, and visionary requirements for the integration of microbiome analysis into clinical routine diagnostics.
- Metagenomics is increasingly used for the culture-independent and largely unbiased characterization of complex bacterial habitats at high resolution. The versatility and decreasing costs of metagenomics make this technology an interesting tool for clinical diagnostics.
- Methodological shortcomings still impede the application of metagenomics in clinical diagnostics.
- Integration of metagenomics into clinical medicine requires accepted and validated strategies for (1) translation into clinical action items; (2) sample collection, preparation, and testing; and (3) data analysis and interpretation. We highlight tasks that are of high priority from a clinical perspective for the useful medical application of metagenomics.

## Introduction

The intestine is one of the most diverse and complex bacterial habitats of the human body, harboring ∼1,000 bacterial phylotypes [1]. Recent studies have associated the human intestinal microbiome (i.e., the collective genomes of all intestinal microbial habitants [2]) with health and disease states, suggesting that metagenomic analysis of the intestinal microbiome could be exploited as a novel diagnostic, prophylactic, or therapeutic strategy in multiple medical specialties. For example, the identification and quantification of opportunistic pathogens in the intestinal microbiome may facilitate risk stratification in immunocompromised patients, such as in critically ill, HIV-infected or immunosuppressed (e.g., organ transplant recipients or individuals with autoimmune disease) patients. Also, the correction of intestinal dysbiosis, the pathologic imbalance of the gut microbiota, may inhibit the development and/or delay the progression of autoimmune diseases [3],[4], metabolic disorders [5], and cancer [6]. The propagation of a healthy intestinal microbiota has even been shown to reduce toxicity and increase effectiveness of cancer therapies in rats [7]. In addition, standard analysis of the human intestinal microbiome in patients may enable the rapid identification of novel emerging infectious pathogens in fecal specimens, for example, in the case of an outbreak of Shiga-toxigenic *Escherichia coli* [8].

Our understanding of the human intestinal microbiome in health and disease has been revolutionized by the development of next generation sequencing and its application to metagenomics, which is the term generally used to summarize culture-independent technologies that allow the characterization of a microbiome [2]. These methods allow for the largely unbiased characterization of complex microbial communities at high resolution, including the detection of novel and uncultivable bacteria, viruses, archaea, and small eukaryotic organisms, even in compartments previously considered to be sterile, such as the urinary bladder [9]. The European MetaHIT project (http://www.metahit.eu) and the US National Institutes of Health Human Microbiome Project (http://www.hmpdacc.org) have set new standards for the in-depth metagenomic characterization of the healthy human microbiota (microorganisms living inside and on humans) from different body habitats [2].

Optimizing patient outcome according to metagenomic information depends on the quality of the available information, options for translation of this information into clinical action, and the effectiveness of communication. Translation of metagenomic knowledge into clinical practice is impeded by several limitations. For example, vast amounts of information are generated by metagenomics, which has to be assorted, interpreted, and communicated to clinicians in a comprehensible format. Most clinical studies have focused on characterizing the human microbiota by its taxonomic composition using 16S rRNA–based bacterial surveys, although similar biological functions may be exerted by unrelated taxa [10]. Establishing a cause–effect relationship or using microbiome profiles as surrogate markers for diseases is accordingly difficult.

## Priorities for the Application of Metagenomics in Clinical Medicine

Strategies still remain to be defined for (1) translation into clinical action items with impact on patient outcome; (2) sample collection, preparation, and testing; and (3) data analysis, interpretation, and communication. Here, we highlight the tasks that are of high priority from a clinical perspective for the useful application of metagenomics in clinical medicine.

## Priority 1: Integration of Metagenomic Information with Other Clinical and Laboratory Sources of Evidence for Translation into Targeted Therapy

Metagenomic information has been associated with specific disorders in several studies. For example, clinical observations have long suggested that the intestinal microbiome plays a critical role in the pathogenesis of inflammatory bowel disease (IBD) (Crohn disease and ulcerative colitis): (1) inflammation in Crohn disease disappears if the involved bowel segment is excluded from the fecal stream and recurs after re-anastomosis with reexposure to intestinal contents [11]; (2) IBD responds at least partially to antimicrobials [12] and some probiotics (live bacteria or yeast preparations) [13]; (3) some studies have shown for IBD a decreased bacterial diversity and a shift from anti-inflammatory commensals to pro-inflammatory pathogens (dysbiosis)—particularly to an overrepresentation of proteobacteria and to a reduction in *Faecalibacterium prausnitzii* and other beneficial butyrate-producing bacteria [14]–[16].

While current evidence strongly suggests that the pathogenesis of IBD could be linked to the intestinal microbiota, important clinical questions remain unanswered. So far, study results analyzing microbiome changes in IBD patients were not controlled for potential confounders such as mucosal inflammation per se [17],[18], accelerated intestinal transit due to diarrhea [19], or medications used for IBD treatment, for example, antibiotics and immunosuppressants [20],[21]. In addition, evidence from animal models still has to be confirmed in human clinical medicine, such as the anti-inflammatory properties of *F. prausnitzii* in chronic intestinal inflammation [22]. Results from clinical studies are sometimes incongruous—initial studies of patients with ulcerative colitis showed a marked benefit from fecal microbiota transplantation (FMT) [23], but other small studies could not confirm this observation [24]. Another study showed that FMT could correct the proposed features of the dysbiotic intestinal microbiota in IBD, such as the increased abundance of proteobacteria, but did not result in significant clinical improvement [24].

Hence, metagenomics approaches have to fulfill several clinical prerequisites to have a significant impact on diagnostic, prophylactic, and therapeutic strategies. A cause–effect relationship between a defined disorder and intestinal microbiome profile has to be established beyond doubt. A clear distinction between intestinal microbiome profiles of disorders (e.g., IBD versus other causes of intestinal inflammation) on the basis of metagenomic information would greatly facilitate diagnostic strategies. Identification of significant confounders of metagenomic information (inflammation, concomitant therapy, diet, etc.) may also help in devising novel prophylactic strategies. Well-directed strategies for the targeted therapy of disorders of the intestinal microbiome have to be developed, and existing ones optimized (e.g., selection of FMT donors according to a target microbiome). For this purpose, longitudinal studies with well-defined intervention and control groups as well as adequate follow-up periods are warranted. Metagenomic information on longitudinal changes in the intestinal microbiome needs to be combined with other clinical and laboratory sources of evidence for translation into targeted therapies.

## Priority 2: Standardization of Diagnostic Procedures in Sample Collection, Preparation, and Testing

Accurate sample collection, preparation, and analysis are of paramount importance for the characterization of the intestinal microbiome in health and disease. Collection of stool samples; collection of gastric, intestinal, or biliary fluid; and endoscopic mucosal biopsies are routine clinical procedures. Next generation sequencing already allows characterization of the microbial composition of a sample (e.g., by 16S rRNA gene region analysis) and of its genetic and functional potential (reviewed in [25],[26]).

Nevertheless, the choice of sample, sampling procedure, and analytical workflow greatly influences the results and thus the clinical utility of metagenomic characterization. Microbiota compositions fluctuate in response to dietary and sanitary habits, age, genotype, sex, ethnicity, and use of antibiotic and other medications [27]–[29]. Sample contamination from other anatomic regions (e.g., from oropharynx to stomach) is difficult to avoid with currently available endoscopic tools [30]. The clinically most significant anatomic locations in relation to a specific intestinal disorder still have to be defined (e.g., fecal sample versus endoscopic biopsy, or sampling of lesions versus surrounding, unaffected mucosa in IBD). Finally, differences in sample preparation, DNA isolation, metagenomic approaches, number of reads analyzed, and sequencing instrument used have a large impact on the final results [27].

Standardization of workflows in metagenomic studies is therefore urgently needed. Sampling methods have to be developed to avoid carryover contaminations. Standards must to be adapted and optimized to specific human cohorts and diseases for a meaningful interpretation of metagenomic information.

## Priority 3: Automation of Data Analysis, Interpretation, and Communication

Analysis and statistical interpretation of the data in a reproducible form are also vital for the translation of metagenomics information into clinical action items [31]. Basically, sequence reads from the sampled DNA are clustered into operational taxonomic units, which are taxonomically classified and compiled into a list of relative operational taxonomic unit abundances for each sample (reviewed in [32]). Next, the whole-community composition can be statistically evaluated and categorized for clinical purposes according to function, prevalence, absence, or alternation of particular bacterial groups. These groups of interest can range from broad taxonomic classes to specific bacterial families or species, such as the two phyla Firmicutes and Bacteroidetes, whose ratio has relevance to obesity [33]; members of the phylum Proteobacteria, whose abundance has been associated with intestinal disease states such as IBD [18]; Clostridia species that induce anti-inflammatory regulatory T-cells [34]; or tumor-inducing *Fusobacterium nucleatum* [35].

Currently, the introduction of metagenomic tools into clinical practice is facing major technical as well as biological obstacles: (1) long analysis times, (2) evolving definitions of reference microbiota, (3) missing standards of analysis methods, algorithms, and databases, (4) lack of well-defined physiological ranges, and (5) missing evidence for cause–effect relationships.

From a technical perspective, a maximum level of automation would facilitate the digest of metagenomic data into clinically meaningful information. Analysis speed is highly dependent on the number of collectively analyzed samples, and the methods and tools used. Filtering and quality improvement steps may require several days, even on medium-sized computing clusters. Hence, rapid data analysis needs a reference microbiome as a reliable standard with which to compare individual samples, reduction of analysis complexity, and, ultimately, integration of analysis algorithms and desktop sequencers into a single package. Furthermore, for meaningful interpretation and communication, results of statistical evaluations should be generated and digested into clinically relevant bits automatically in the same sequencing unit, and communicated as an analysis report to the physician within a few hours. A crucial biological point is the definition of physiological ranges of gut microbiota parameters, which are highly variable between ethnic groups, geographic locations, and different diets [36]. For the definition of reference values, representative samples from the local healthy population have to be analyzed for the relative abundance of taxonomic groups or ratios between groups, combined with relevant clinical data (see the Human Microbiome Project and the American Food Project [http://humanfoodproject.com]). This information would also provide the basis for establishing cause–effect relationships. Finally, reference values have to be updated continuously and integrated into analysis algorithms for effective translation of evolving insight into intestinal microbiota into clinical practice.

## Outlook

The establishment of characteristic and thoroughly validated signatures of the intestinal microbiome allows the development of new prophylactic, therapeutic, and prognostic strategies for beneficial and targeted modification of the patient's intestinal microbiome. Most metagenomic tools required for addressing these important questions are already available, standard operating tools are under development (see the Human Microbiome Project), and insight into the human microbiome is evolving rapidly (Box 1). Modern, high-resolution, and high-throughput analysis of complex bacterial communities in clinical samples has the potential to revolutionize clinical practice. As a prerequisite, target conditions must be specified, conclusively linked with characteristic signatures of the intestinal microbiome, and thoroughly validated. In addition, sample collection, preparation, testing, analysis, and result interpretation must be standardized and widely automated, and costs per sample and turnaround times significantly reduced. The integration of metagenomic analysis into clinical diagnostics will very likely open whole new avenues to the treatment of intestinal as well as extra-intestinal diseases.

**Box 1. Five Key Papers on the Translation of Metagenomics into Clinical Practice**

1. **Qin J, Li R, Raes J, Arumugam M, Burgdorf KS, et al. (2010) A human gut microbial gene catalogue established by metagenomic sequencing. Nature 464: 59–65.** This study reports a large-scale approach to characterizing the functionality of the intestinal microbiota by cataloging human gut microbial genes, which is a prerequisite for defining health and disease states in terms of the microbiome.
2. **Human Microbiome Project Consortium (2012) Structure, function and diversity of the healthy human microbiome. Nature 486: 207–214.** This project is a trendsetting approach to establishing comprehensive metagenomic datasets of (healthy) body habitats as reference datasets and to lay the foundation for the translation of metagenomic research into diagnostic applications.
3. **Kump PK, Gröchenig HP, Lackner S, Trajanoski S, Reicht G, et al. (2013) Alteration of intestinal dysbiosis by fecal microbiota transplantation does not induce remission in patients with chronic active ulcerative colitis. Inflamm Bowel Dis 19: 2155–2165.** This was one of the first attempts not only to use FMT but also to characterize the procedure and the outcome by metagenomics.
4. **Ley RE, Turnbaugh PJ, Klein S, Gordon JI (2006) Microbial ecology: human gut microbes associated with obesity. Nature 444: 1022–1023.** This study links the metagenomics pattern of the human intestinal microbiome to a clinical disorder and is therefore of importance for therapeutic approaches.
5. **Navas-Molina JA, Peralta-Sánchez JM, González A, McMurdie PJ, Vázquez-Baeza Y, et al. (2013) Advancing our understanding of the human microbiome using QIIME. Methods Enzymol 531: 371–444.** This study describes one of the common interactive analysis tools for microbiome analysis currently used by many researchers, which might be used in the future for standardizing data analysis.

## Abbreviations

FMT: fecal microbiota transplantation
IBD: inflammatory bowel disease
